# Monocarboxylate Transporter 4 Facilitates Cell Proliferation and Migration and Is Associated with Poor Prognosis in Oral Squamous Cell Carcinoma Patients

**DOI:** 10.1371/journal.pone.0087904

**Published:** 2014-01-30

**Authors:** Jiang Zhu, Yu-Nong Wu, Wei Zhang, Xiao-Min Zhang, Xu Ding, Huai-Qi Li, Meiyu Geng, Zuo-Quan Xie, He-Ming Wu

**Affiliations:** 1 Institute of Stomatology, Nanjing Medical University, Nanjing, PR China; 2 Department of Oral and Maxillofacial Surgery, Stomatological Hospital of Jiangsu Province, Nanjing, PR China; 3 State Key Laboratory of Drug Research, Shanghai Institute of Materia Medica, China Academy of Sciences, Shanghai, China; China Medical University, Taiwan

## Abstract

Monocarboxylate transporter 4 (MCT4) is a cell membrane transporter of lactate. Recent studies have shown that MCT4 is over-expressed in various cancers; however, its role in cancer maintenance and aggressiveness has not been fully demonstrated. This study investigated the role of MCT4 in oral squamous cell carcinoma (OSCC), and found that it is highly expressed in OSCC patients by using immunohistochemistry. Moreover, this over-expression of MCT4 was closely associated with tumor size, TNM classification, lymphatic metastasis, distant metastasis and tumor recurrence, and also poor prognosis. To further study mechanisms of MCT4 *in vitro*, we used small-interfering RNA to silence its expression in OSCC cell lines. The results showed that knock-down of MCT4 decreased cell proliferation, migration, and invasion. The inhibition of proliferation was associated with down-regulation of p-AKT and p-ERK1/2, while decreased cell migration and invasion may be caused by down-regulation of integrin β4-SRC-FAK and MEK-ERK signaling. Together, these findings provide new insight into the critical role of MCT4 in cell proliferation and metastasis in OSCC.

## Introduction

Oral squamous cell carcinoma (OSCC) is the sixth most common malignant cancer worldwide, with approximately 300 thousand patients every year. [1] OSCC is characterized by severe progression, partnered with a high potential for both nodal metastasis and locoregional invasion. [2] Despite recent advances in surgery, radiotherapy, and chemotherapy, the 5-year survival rate of this disease has remained at 50% for the past 30 years.[3] Thus, further research is required to understand the mechanisms driving the progression of OSCC and potentially identify targets for improved therapeutic intervention. It has been reported that nodal and distant metastasis in head squamous cell carcinoma is associated with high tumor lactate concentration.[4], [5] Moreover, most tumor cells generate significantly greater levels of lactate when compared with that of normal human oral keratinocytes.[6] Indeed, in malignant carcinoma, a large amount of metabolic energy is required to meet its demand for uncontrolled proliferation.[7] Therefore, cancer cells rapidly convert the majority of glucose into lactate to obtain sufficient ATP, even in the presence of sufficient oxygen, to support mitochondrial oxidative phosphorylation. This phenomenon is known as the “Warburg effect” and leads to the production of a large amount of acids, mostly lactate.[8] However, lactate accumulation in cancer cells induces apoptosis and therefore it must be transported out of the cells.[9]

It is widely known that monocarboxylate transporter 4 (MCT4) transports lactate out of cells, which is very important for the acid-resistant phenotype of cancer cell.[10] Moreover, by exporting newly formed lactate, MCT4 maintains the continuous conversion of pyruvate to lactate and therefore continuous glycolysis.[8] However, efflux of lactate leads to an acidic tumor environment, which has been demonstrated to enhance cellular motility.[11], [12], [13] Besides being an end product, lactate exported by MCT4 may also be consumed by oxygenated oxidative cancer cells located in the peripheral part of the cancer. [14], [15], [16] Thus, in addition to its role, as the transporter responsible for lactate efflux from cancer cells that is important in tumor metabolism, MCT4 is also involved in the lactate-induced malignant behavior of cancer cells.

Indeed, high expression of MCT4 was recently shown to be closely associated with increased cellular motility and invasive potential in *in vitro* models of breast and lung cancer, [9], [17] and has been associated with poor prognosis in prostate and colorectal cancer.[18], [19] MCT4 has also been described as an effective metabolic target to reverse the Warburg effect compared with the ubiquitously expressed MCT1 in clear cell renal cell carcinoma.[20] However, the role of MCT4 in OSCC and the underlying molecular mechanisms involved in mediating its effects are currently unknown.

In the present study, we examined the expression of MCT4 in clinical samples and its correlation with clinical and pathological parameters. Then, we explored the function of MCT4 in cell lines by modulating its levels using RNA interference, and provide evidence that MCT4 is closely related with cell proliferation, migration, and invasion ability in OSCC. Indeed, we found that MCT4 mediates cell proliferation *via* two major proliferation pathways, AKT and MEK-ERK. In addition, we found that the decreased cell migration and invasion mediated by the loss of MCT4 could also be achieved by disabling the intracellular link of integrin β4 to SRC-FAK and MEK-ERK signaling. Thus, this study firstly describes an important role for MCT4 in cell proliferation and invasive behavior in OSCC.

## Materials and Methods

### Ethics Statement

Human paraffin embedded tissue samples were collected from 99 patients (59 were male and 40 were female), who were examined and treated for OSCC at the Stomatological Hospital of Jiangsu Province, Nanjing, China. Written informed consents from these patients were obtained for use of the tissue samples and for follow-up interviews in research. The study was conducted in accordance with the guidelines in the Declaration of Helsinki. Ethical review board (Committee of ethics of Nanjing Medical University) approved the use of human paraffin embedded tissues and OSCC cell lines in vitro. All patients underwent radical resection without any previous radiation or chemotherapy. Patients' clinical data are shown in Table 1. Tumor grade was classified as poorly differentiated, moderately differentiated, and well differentiated. The pathological stage was defined according to the American Joint Committee on Cancer (AJCC) TNM staging system. Both tumor grade and pathological stage were evaluated by two pathologists independently. Primary tumor sites included tongue (n = 36), gingiva (n = 20), buccal mucosa (n = 28), mouth floor (n = 3), palate (n = 7), and jaw (n = 5). Follow-up data were collected through direct interviews with patients or their relatives. At the time of data collection, 24 patients (24%) showed evidence of disease recurrence and 32 patients (32%) had died of the disease.

**Table 1:** Clinical and pathological data of the patients.

Category	Subcategory	Number of patients (%)
Gender	Male	59(59.6%)
	Female	40(40.4%)
Age(y)	≤60	51(51.5%)
	>60	48(48.5%)
Histologic grade	Well differentiation	56(56.6%)
	Moderate differentiation	36(36.4%)
	Poor differentiation	7 (7.0%)
Tumor size(cm)	≤4	71(71.7%)
	>4	28(28.3%)
TNM classification	Class 1–2	48(48.5%)
	Class 3–4	51(51.5%)
Lymphatic metastasis	Positive	42(42.4%)
	Negative	57(57.6%)
Distant metastasis	Positive	10(10.1%)
	Negative	89(89.9%)
Tumor recurrence	Positive	24(24.2%)
	Negative	75(75.8%)

### Immunohistochemistry (IHC)

MCT4 antibody (sc-376140; Santa Cruz Biotechnology, Santa Cruz, CA, USA) was applied for IHC detection of the MCT4 protein in OSCC tissue sections in accordance with the avidin-biotin-peroxidase complex principle (R.T.U. Vectastain Elite ABC Kit [Universal]; Vector Laboratories, Burlingame, CA, USA). The MCT4 antibody was diluted 1∶200 according to the manufacturer's instructions. Negative controls were included by incubating tissue sections with normal mouse serum or phosphate-buffered saline. All sections were counterstained with hematoxylin.

### Quantification and evaluation of MCT4 expression detected by IHC

MCT4 expression was evaluated semi-quantitatively as the total MCT4 immunostaining score, which was calculated as the sum of a proportion score and an intensity score. Briefly, the fraction of positive staining cells were defined as the proportion score cells where score 0, <5%; score 1, 5–10%; score 2, 10–50%; score 3, 50–75%; and score 4, >75%. The staining intensity was evaluated as score 0, no staining signal; score 1, weak positive signal; score 2, moderate positive signal; and score 3, strong positive signal. Finally, a total expression score was obtained that ranged from 0 to 7. The over-expression of MCT4 was defined as a total expression score ≥6. Evaluation was performed independently by two observers and the average of two readings was used for statistical analysis.

### Cell lines

The human OSCC cell lines Cal27, SCC9, and SCC25 were purchased from American Type Culture Collection. Human OSCC cell line HN4, HN6, and HN12 were generously donated by the Shanghai Ninth People's Hospital (Shanghai, China), which had been used in previous study [21]–[23]. HN4, HN6, HN12, and Cal27 were maintained in Dulbecco's Modified Eagle's Medium (DMEM) (Gibco, Grand Island, NY, USA), and SCC9 and SCC25 were maintained in DMEM-F12 (Gibco). All cell lines were supplemented with 10% fetal bovine serum and maintained at 37°C in a 5% CO_2_ humidified incubator.

### Small interfering RNA (siRNA) and transient transfection

siRNA was used to silence the MCT4 gene. MCT4 siRNA: siMCT4#1, 5′-CCUACUCCGUCUACCUCUUTT-3′ (sense) and 5′-AAGAGGUAGACGGAGUAGGTT-3′ (antisense); siMCT4#2, 5′-GGCAACUUCUUCUGCAUUATT-3′ (sense) and 5′-UAAUGCAGAAGAAGUUGCCTT-3′ (antisense) were commercially synthesized by Guangzhou RiboBio Corporation (Guangzhou, China). A scrambled sequence siRNA (siNCtrl) was also used as a negative control (Guangzhou RiboBio Corporation, Guangzhou, China). Lipofectamine™-RNAimax (Invitrogen, Carlsbad, CA, USA) was used to optimize siRNA transfection according to the manufacturer's instructions. Briefly, lipofectamine and siRNA were diluted separately in serum-free Opti-MEM (Gibco) and incubated for 5 min at room temperature. Then, the two solutions were gently mixed and incubated together for 20 min. After incubation, the complex was added to the plated cells. Three days later, or more, cells were further assayed as detailed below.

### Real-time PCR

Total RNA was extracted form cells using Trizol Reagent following the manufacturer's protocols (Invitrogen, Carlsbad, CA, USA). cDNA was synthesized from 2.5 µg total RNA by PrimeScript RT Master Mix Perfect Real-Time (TaKaRa, Tokyo, Japan). Real-time PCR was performed by using a TaqMan ABI 7300 Sequence Detection System (PE Applied Biosystems, Foster City, CA). MCT1, MCT4 and GAPDH mRNAs were detected, using SYBR Green PCR Master Mix Reagent (SYBR Premix Ex Taq kit; TaKaRa). The primer sequences were as follows: MCT1, 5′-TTTCTTTGCGGCTTCCGTTGTTG-3′ (forward) and 5′-TCAATTTACCCTTCAGCCCCATGG-3′ (reverse); MCT4, 5′-TTTTGCTGCTGGGCAACTTCTTCTG-3′ (forward) and 5′-TCACGTTGTCTCGAAGCATGGGTTT-3′ (reverse). The results of real-time PCR were represented as Ct values, where Ct is a fraction defined as the cycle number at which the sample fluorescent signal passes a given threshold above the baseline. The ΔCt is the difference in the Ct values derived from specific genes after comparison with GAPDH.

### Medium lactate measurement

Eighty-four hours after transfection of siRNA into OSCC cells, medium was replaced with serum-free medium, and lactate secretion was measured after a further 12 h using the Lactate assay kit from Jianchen Bioengineering Institute (Nanjing, China). Meanwhile, cell lysates were collected for total protein quantification. Lactate levels were normalized by total protein.

### CCK8 assay

Cells were seeded into 96-well plates at the density of 2000 cells per well in 80 µl medium and incubated for 12 h, then cells were treated with 20 µl lipofectamine-siRNA complexes, which contained 2 nM siRNA, cells treated with scrambled siRNA were used as a negative control. And 96 h after transfection, cell proliferation rate was measured by adding 10 µl of CCK8 solution (Dojindo, Tokyo, Japan) to each well, followed by incubation at 37°C for 2 h. Absorbance was measured by SPECTRA MAX190 spectrophotometry (Sunnyvale, CA, USA) at 450 nm. In each assay, six parallel wells were included, and the results were collected as the mean of three independent experiments.

### Cell proliferation sulforhodamine B (SRB) assay

Cells were seeded into 96-well plates at the density of 2000 cells per well in 80 µl medium and incubated for 12 h, then cells were treated with 20 µl lipofectamine-siRNA complexes, which contained 2 nM siRNA, for 96 h. Cells treated with scrambled siRNA were used as a negative control. After incubation, cells were fixed with 10% trichloroacetic acid for 12 h at 4°C and then stained with 0.4% SRB diluted in 1% acetic acid for 20 min followed by washing with 1% acetic acid. The resulting precipitates were dissolved in 10 mM Tris-HCl and measured by SPECTRA MAX190 spectrophotometry (Sunnyvale, CA, USA) at 560 nm. In each assay, six parallel wells were included, and the results were collected as the mean of three independent experiments.

### Migration and invasion assay

Cell migration assays were conducted using 24-well Corning chambers with the non-coated membrane (24-well insert; pore size, 8 µm; Corning, Corning, NY, USA) while invasion assays were conducted using 24-well Corning chambers coated with 1∶10 serum-free DMEM diluted in matrigel. Briefly, MCT4 siRNA, or control, transfected HN6 cells were seeded into upper inserts (30,000 cells per insert) in serum-free DMEM. Outer wells were filled with 600 µl DMEM containing 10% FBS to act as the chemoattractant. Cells were allowed to migrate and invade for 12 h and 18 h, respectively. The migrated or invaded cells on the bottom face of the filter were fixed in 90% ice-cold ethanol and stained with 8% crystal violet. The entire membrane was photographed by light microscopy. Each assay was conducted in duplicate and repeated three times.

### Wound healing assay

Cells (40,000 cells per well) were seeded in 24-well plates and transfected with siRNA for 96 h. After formation of a complete monolayer, scratches were made within the monolayer using a standard 200-µl plastic tip. Migration and cell movement throughout the wound area was observed under a phase-contrast microscope at the same spot after 8 h. The percentage of filled wound area was calculated using the following equation: wound filled (%)  =  (original wound area − remaining wound area)/original wound area ×100. Each assay was conducted in triplicate and repeated three times.

### Western blot

Cell lysates were applied and subjected to 7.5–10% PAGE and transferred to nitrocellulose filter membranes. Membranes were then blocked for 1 h in 5% non-fat dry milk diluted in TBST (10 mM Tris–HCl and 0.05% Tween 20). The membranes were then incubated with primary antibodies overnight at 4°C, followed by incubation with appropriate secondary antibodies for 2 h at room temperature. The primary antibodies included mouse monoclonal anti-MCT4 (1∶500; Santa Cruz, CA, USA), mouse monoclonal anti-integrin β4 (1∶500; Santa Cruz Biotechnology), rabbit monoclonal anti-p-FAK Tyr397/925 (1∶1000; Cell Signaling, Danvers, MA, USA), rabbit monoclonal anti-FAK (1∶1000; Cell Signaling), rabbit monoclonal anti-p-SRC (1∶1000; Cell Signaling), rabbit monoclonal anti-SRC (1∶1000; Cell Signaling), rabbit monoclonal anti-p-MEK1/2 (1∶1000; Cell Signaling), rabbit polyclonal anti-MEK1/2 (1∶1000; Santa Cruz, CA, USA), mouse monoclonal anti-p-ERK1/2 (1∶1000; Cell Signaling), rabbit monoclonal anti-ERK1/2 (1∶1000; Cell Signaling), rabbit monoclonal anti-p-AKT (1∶1000; Cell Signaling), rabbit monoclonal anti-AKT (1∶1000; Cell Signaling), rabbit monoclonal anti-E-cadherin (1∶1000; Epitomics, Burlingame, CA, USA), rabbit monoclonal anti-N-cadherin (1∶1000; Epitomics), rabbit polyclonal anti-vimentin (1∶1000; Epitomics), rabbit polyclonal anti-GAPDH (1∶5000; Epitomics), and rabbit monoclonal anti-β-actin (1∶1000; Epitomics). The membranes were next incubated in the dark with ECL or Duro or Femto (Thermo Fisher Scientific (Bremen) GmbH, Bremen, Germany) for chemiluminescence detection. Signals were detected using enhanced chemiluminescence detection reagents (GE Healthcare, Waukesha, WI, USA).

### Statistical analysis

The clinical-pathological and IHC data were analyzed with the SPSS statistical software (version 17.0; SPSS Inc, Chicago, IL, USA). The statistical significance between all these comparisons was examined using the Chi-square analysis or the Fisher exact test. Survival curves were made by the means of the Kaplan–Meier method and compared by the long-rank test. And the significant of variables for survival was analyzed using the Cox proportional hazards model in a multivariate analysis. In vitro data were analyzed by One-way ANOVA method, a value of p<0.05 was considered to be statistical significance.

## Results

### Correlation between MCT4 expression and clinical and pathological parameters

**Figure 1:**
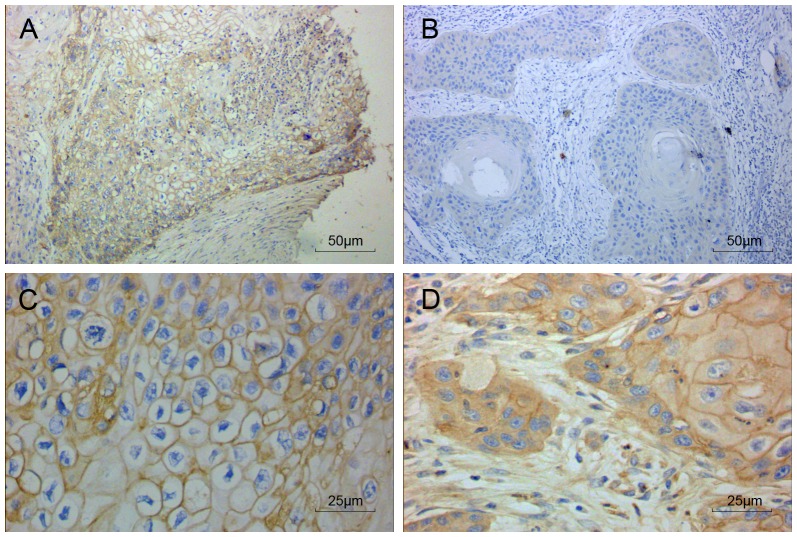
MCT4 expression in oral squamous cell carcinoma by immunohistochemistry. Representative images of tumor and associated stroma (A, C–D) as well as adjacent normal tissue (B) were stained for MCT4 (brown). Sections were counterstained with hematoxylin (blue). 200× (A–B) and 400× (C–D) magnifications are shown.

Expression of MCT4 protein was examined in a total of 99 OSCC patient samples by immunohistochemical staining. As shown in Figure 1, MCT4 was mainly expressed in cell membranes, in support of its role as a protein that mediates the transmembrane transport of lactate. MCT4 was also expressed in the cytoplasm of some patients (Figure 1D).

The correlation between MCT4 expression and various clinical and pathological characteristics was also analyzed. As shown in Table 2, MCT4 expression was found to be positively correlated with tumor size (p = 0.006), TNM classification (p<0.001), lymphatic metastasis (p<0.001), distant metastasis (p = 0.009), and tumor recurrence (p = 0.001), while no significant correlation was found between MCT4 expression with gender, age, alcohol and cigarette abuse, or tumor grade.

**Table 2:** Association between OSCC clinical-pathological parameters and MCT4 expression.

		Number of patients (%)		
Category	Subcategory	MCT4 low expression	MCT4 high expression	p value
Gender	Male	30 (30.3%)	29 (29.3%)	0.059
	Female	28 (28.3%)	12 (12.1%)	
Age(y)	≤60	31 (31.3%)	20 (20.2%)	0.651
	>60	27 (27.3%)	21 (21.2%)	
Alcohol and cigarette abuse	positive	15 (15.2%)	5 (5.0%)	0.097
	negative	43 (43.4%)	36 (36.4%)	
Tumor size(cm)	≤4	49 (49.5%)	22 (22.2%)	0.006*
	>4	9 (9.1%)	19 (19.2%)	
Histological grade	Well	34 (34.4%)	22 (22.2%)	0.95
	Moderate	20 (20.2%)	16 (16.2%)	
	Poor	4 (4.0%)	3 (3.0%)	
TNM classification (1–4)	Class 1	9 (9.1%)	3 (3.0%)	<0.001*
	Class 2	27 (27.3%)	9 (9.1%)	
	Class 3	19 (19.2%)	13 (13.1%)	
	Class 4	3 (3.0%)	16 (16.2%)	
Lymphatic metastasis	Positive	16 (16.2%)	26 (26.3%)	<0.001*
	Negative	42 (42.4%)	15 (15.1%)	
Distant metastasis	Positive	2 (2.0%)	8 (8.1%)	0.009*
	Negative	56 (56.6%)	33 (33.3%)	
Tumor recurrence	Positive	7 (7.1%)	17 (17.2%)	0.001*
	Negative	51 (51.5%)	24 (24.2%)	

* Statistically significant difference

### MCT4 expression is highly correlated with poor prognosis in OSCC patients

**Figure 2:**
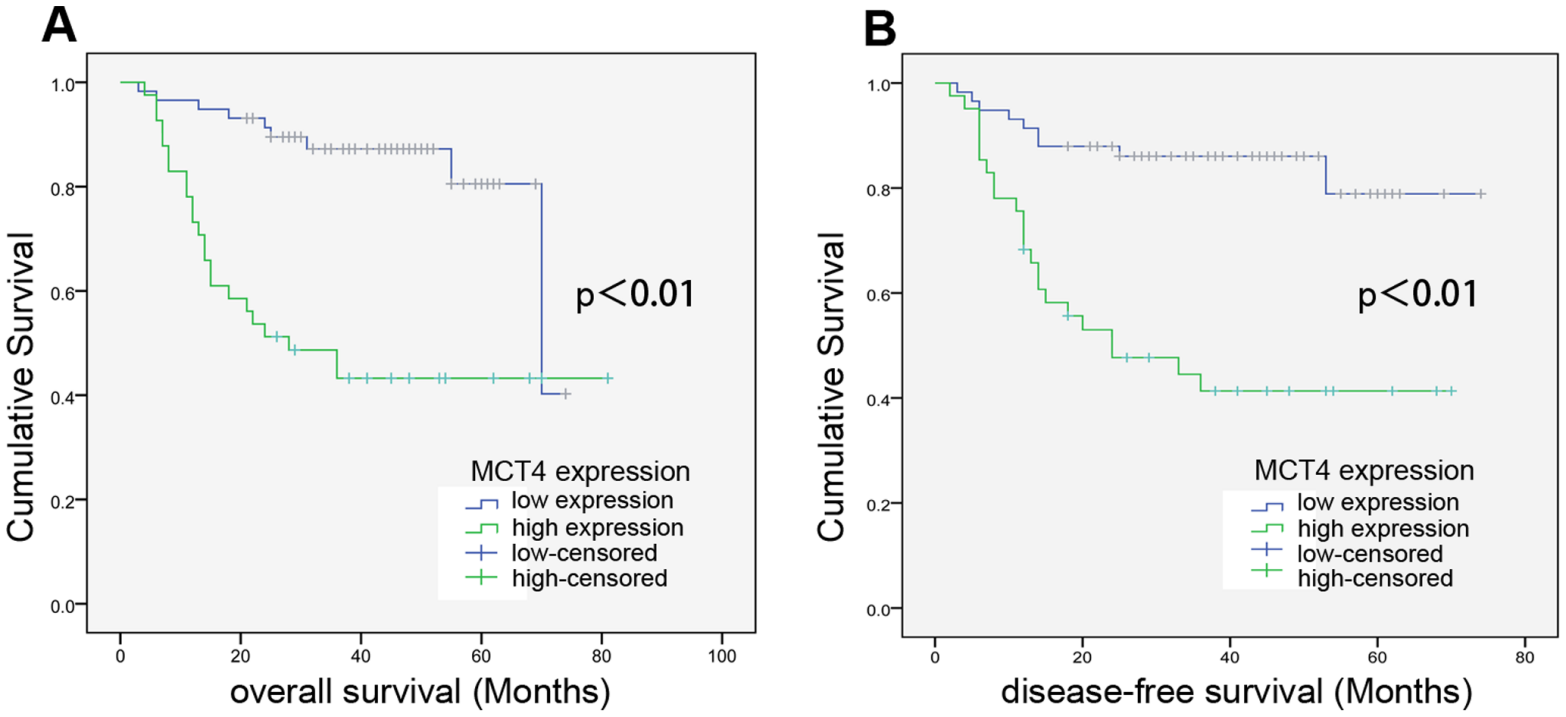
MCT4 expression significantly correlates with reduced OSCC patient survival. Overall survival (A) and disease-free survival (B) in OSCC patients with low- and high expression levels of MCT4, as indicated, were assessed by Kaplan–Meier analysis.

The correlation of MCT4 expression with prognosis was analyzed by the Kaplan–Meier method. As shown in Figure 2, MCT4 expression showed significant correlation with both overall survival (OS) (p<0.001) and disease-free survival (DFS) (p<0.001) in OSCC patients. Patients with high MCT4 expression showed significantly reduced survival time compared with that of those with low MCT4 expression, indicating that MCT4 is an effective prognosis predictor for OSCC patients.

Next, multivariate survival analysis was performed to evaluate the independent prognostic factor of outcomes including gender, age, histological grade, tumor size, TNM classification, and MCT4 expression (Table 3). In addition to TNM classification, which was found to be an independent prognosis factor of OS and DFS, MCT4 was also an independent prognosis factor of OS and DFS in OSCC patients, with a much lower p value (p = 0.002 and 0.003, respectively) as compared with TNM classification (p = 0.008 and 0.012, respectively). These data suggest that the expression of MCT4 is more strongly correlated with the poor prognosis of OSCC patients than TNM classification.

**Table:** Table 3. Summary of survival analysis by multivariate Cox regression analysis.

Parameter		Overall survival			Disease-free survival		
Category	Subcategory	p	Exp(B)	95% CI	p	Exp(B)	95% CI
Gender	Male vs Female	0.707	0.861	0.395–1.876	0.725	0.870	0.399–1.896
Age(y)	≤60 vs >60	0.708	0.864	0.403–1.853	0.814	0.913	0.427–1.952
Histological grade	Well vs Poor	0.075	1.554	0.956–2.524	0.103	1.507	0.920–2.468
Tumor size (cm)	≤4 vs>4	0.128	0.496	0.201–1.223	0.188	0.547	0.223–1.343
TNM classification	I–II vs III–IV	0.008	2.428	1.256–4.694	0.012	2.301	1.197–4.421
MCT4 expression	Low vs high	0.002	3.641	1.599–8.293	0.003	3.423	1.506–7.781

### Knock-down of MCT4 leads to decreased lactate release

**Figure 3:**
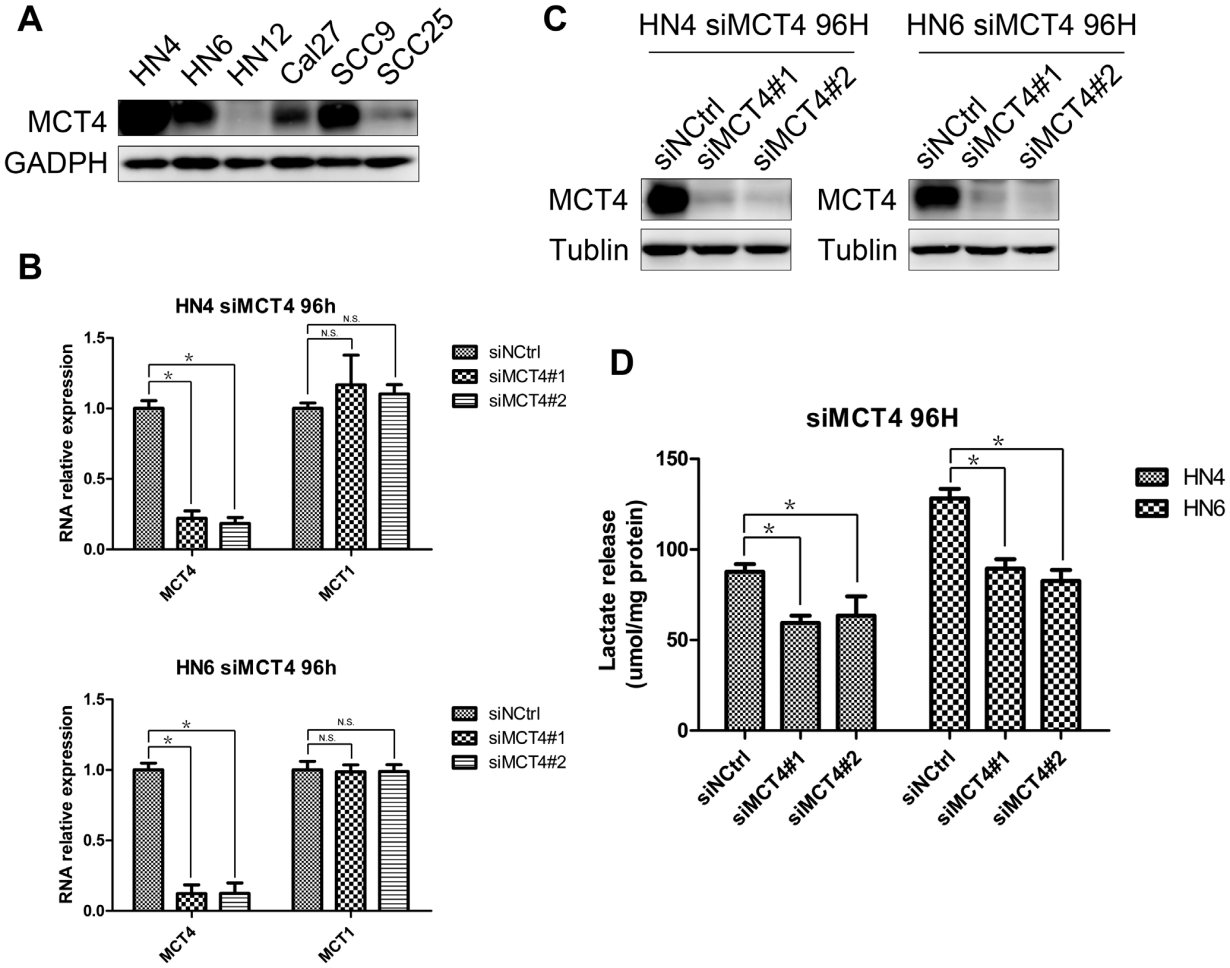
MCT4 expression in OSCC cell lines and the effects of its silencing on lactate release. A. Western blot analysis was used to assess MCT4 protein expression in six OSCC cell lines. B. HN4 and HN6 were transfected with two siRNAs (siMCT4#1 and #2) or a negative control sequence (siNCtrl) for 96 h, then MCT4 mRNA and MCT1 mRNA were analyzed and normalized to GAPDH mRNA, by using real-time PCR. Data represent mean and standard deviation of duplicates from three independent experiments. * p<0.05, and N.S. indicates “not significant”. C. Western blot analysis was used to assess knock-down of MCT4 96 h after transfection of HN4 and HN6 with two siRNAs (siMCT4#1 and #2) or a negative control sequence (siNCtrl). D. Lactate release was measured after knock-down of MCT4 in HN4 and HN6 cell lines. Lactate levels were normalized by total protein. Data represents as mean and standard deviation. * p<0.05.

To assess the cellular expression level of MCT4, its protein expression was evaluated in six OSCC cell lines by western blotting. MCT4 was strongly expressed in HN4, HN6, and SCC9, moderately expressed in Cal27 and SCC25, and exhibited low expression in HN12 (Figure 3A). It is known that MCT4 transports lactate out of the cell while MCT1 controls the entry of lactate into the cell. As MCT4 was knocked down by siRNA, after 96 h, the mRNA levels of MCT4, as well as MCT1 were measured by real-time RCR. It was shown in Figure 3B that both siMCT4#1 and siMCT4#2 showed dramatically decreased MCT4 mRNA levels, while expression of MCT1 was not affected after the knock-down of MCT4, which showed the MCT1 was not compensate for the reduction of MCT4. Western blot result also confirmed the knock down of MCT4 in both HN4 and HN6 cells (Figure 3C). As MCT4 transports lactate out of cells, lactate release was measured to evaluate MCT4 function. When MCT4 was knocked down, lactate release was significantly decreased, as compared with that of the scramble siRNA control (Figure 3D).

### Silencing of MCT4 decreased cell proliferation

**Figure 4:**
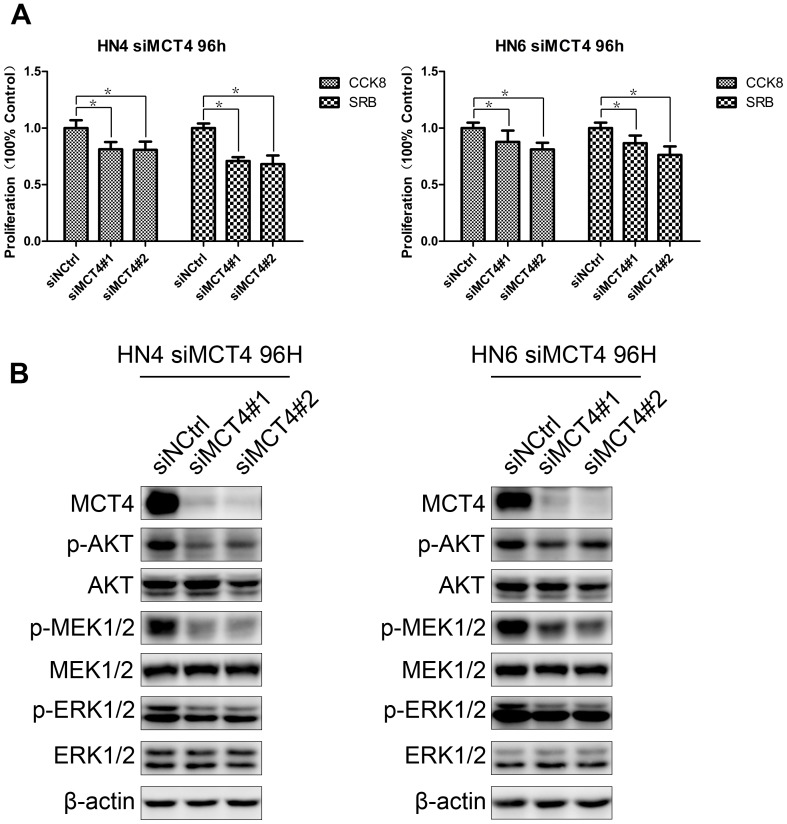
Silencing of MCT4 decreases cell proliferation. A. Proliferation of HN4 and HN6 cells was measured 96(siMCT4#1 and siMCT4#2) or a negative control (siNCtrl) by the CCK8 and SRB methods. Data represent mean and standard deviation of duplicates from three independent experiments. *: p<0.05. B. Lysates from identically treated cells were used to examine the protein expression levels of key proliferation-associated signaling molecules, as indicated, by western blot. MCT4 knock-down was also confirmed. β-actin was used as a loading control.

To explore the close association between MCT4 expression and tumor size that was observed in our clinical samples, we knocked down expression of MCT4 and tested whether cell proliferation was changed. HN4 and HN6, which highly expressed MCT4, were selected as the cell models. Consistent with the observation in clinical patients, cell proliferation was significantly decreased 96 h after MCT4 knock-down in HN4 cells, when compared with that of the control group. The same phenomenon was also observed in HN6 (Figure 4A). To dissect the mechanisms underlying MCT4 knock-down-mediated inhibition of cell proliferation, signaling molecules closely associated with cell proliferation, including two major proliferation pathways, AKT and MEK-ERK1/2, were examined (Figure 4B). We observed that after MCT4 knock-down, p-AKT, p-MRK, and p-ERK1/2 protein expression levels were all down-regulated compared with those of the control group. Thus, this result shows that MCT4 can influence two main cell proliferation pathways, AKT and MEK-ERK.

### MCT4 silencing impaired cell migration and invasion

**Figure 5:**
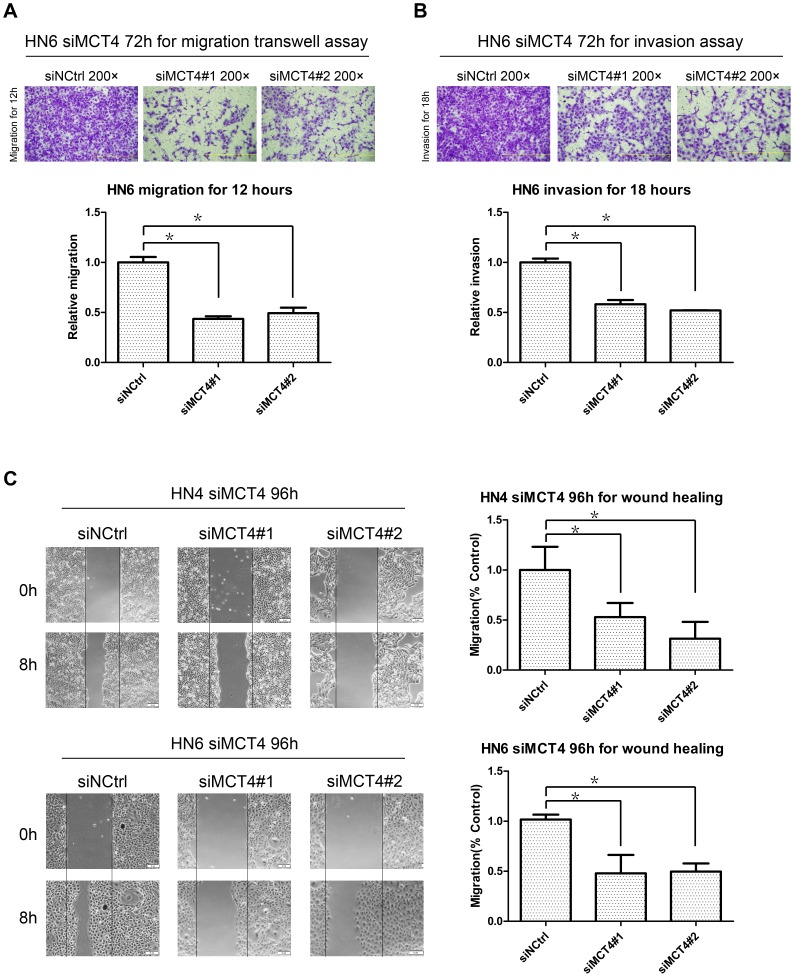
Effect of MCT4 knock-down on migration and invasion. A–B. Migration (A) and invasion assays (B) were performed at the indicated time points using HN6 cells transfected with MCT4 siRNA (#1 and #2) or a negative control (siNCtrl). Top: Representative images of crystal violet stained migratory and invasive cells. Bottom: Relative quantification of images by OD measurement of solubilized crystal violet dye. Data represent mean + SD of duplicates from three independent experiments. *: p<0.05. C. Wound-closure assays were performed with both HN4 and HN6 cells transfected with MCT4 siRNA (siMCT4#1 and siMCT4#2) or a negative control (siNCtrl). The scratch was made with a standard 200-µl plastic tip and set as time zero (0H). The wound area was observed with a phase-contrast microscope at the same spot after 8 h (8H). Left: Representative images. Right: The percentage of filled wound area was calculated and is represented as mean + SD relative to the control. *: p<0.05.

As MCT4 expression was closely related with lymphatic metastasis and distant metastasis in the clinical samples, we examined the effect of MCT4 on cell migration and invasion *in vitro*. Using the migration and Matrigel invasion assays (Figure 5A and 5B), we found that HN6 cells with MCT4 knock-down exhibited significantly reduced invasion as compared with cells treated with scramble siRNA. In the wound-healing assay (Figure 5C), wound closure rates decreased dramatically in both HN4 and HN6 after MCT4 was silenced.

**Figure 6:**
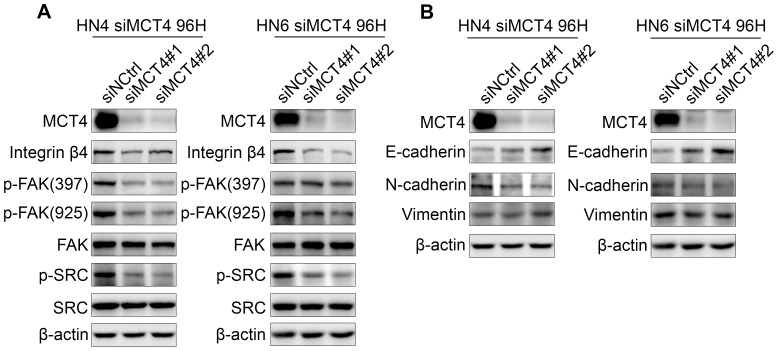
Molecular mechanisms underlying the restrained migration and invasion induced by knock-down of MCT4. A. Protein expression levels of MCT4 and that of key molecules involved in the regulation of cell adhesion, as indicated, were assessed 96(siMCTR#1 and siMCT4#2) or negative control (siNCtrl) by western blot. B. Protein expression levels of key EMT-associated molecules, as indicated, were assessed in identically treated samples by western blot. β-actin was used as a loading control (A–B).

To dissect the molecular mechanisms underlying the restrained migration and invasion induced by loss of MCT4, we examined intracellular signaling by measuring the expression levels of key molecules, such as integrins, FAK, SRC, and those of the MEK-ERK axis, that are involved in the regulation of adhesion, motility, and invasion, as well as growth and survival. Keeping this in mind, we found that in both HN4 and HN6 cells, MCT4 silencing induced depletion of integrin β4 and, in turn, blocked the phosphorylation of FAK-Tyr397, FAK-Tyr925, SRC, MEK, and ERK1/2 without affecting the total levels of FAK, SRC, MEK, or ERK1/2 (Figure 4B and 6A).

There is evidence that phosphorylation at FAK-Y925 is the major SRC-specific phosphorylation event associated with integrin adhesion dynamics and E-cadherin deregulation during SRC-induced epithelial–mesenchymal transition (EMT). [24] As loss of E-cadherin is a key biomarker of EMT, [25] we checked its expression in MCT4 knock-down cells and found that E-cadherin was increased and N-cadherin was decreased both in HN4 and HN6 cells, while vimentin, another EMT marker, was not significantly changed (Figure 6B). These data further support the notion that silencing of the MCT4, together with decreased migration and invasion ability, might also reverse EMT by inhibiting the activity of the integrin-FAK-SRC signaling complex.

## Discussion

This current study demonstrates a critical role for MCT4 in cell proliferation and metastasis of OSCC. We found that strong expression of MCT4 in clinical samples correlates with clinical and pathological parameters. While depletion of MCT4 in OSCC cell lines markedly hinders cell proliferation, migration, and invasion ability. Moreover, MCT4 was found to mediate cell proliferation via two major proliferation pathways, AKT and MEK-ERK, and its effect on cell migration and invasion could be achieved by disabling the intracellular links between integrin β4 to SRC-FAK and MEK-ERK.

MCT4 is a membrane protein that is mostly associated with the export of lactate in cells with high glycolytic rates related to hypoxic energy production.[26] Early malignant tumors occur in an avascular environment, whereby cancer cells depend on glucose and oxygen diffusion through both blood vessels and the basement membrane to fulfill their major metabolic needs.[27], [28] Hence, as early hyperplastic lesions develop quickly and become further than a few cell layers beyond the blood vessel and basement membrane, regional hypoxia will occur, limiting cell growth. This intermittent hypoxia leads to the selection of cells with anaerobic glycolysis, which produces sufficient energy to support further cell growth.[27], [28], [29] Large amounts of glucose are rapidly converted into lactate in these glycolytic cells. Thus, an acid-resistant phenotype is an essential condition for cancer cells to survive.[8] Hence, different pH regulating systems have developed in cancer cell plasma membranes, including those modulated by MCT1, MCT4, Na+/H+ exchanger 1, carbonic anhydrase IX, and anion exchanger 1. Although MCT4 is not the major H+ transporter, it remains one of the most important as it performs a double role in the adaptation to hypoxia: export of lactate, essential to the hyper-glycolytic phenotype; and pH regulation, important to the acid-resistant phenotype.

Strong MCT4 expression has been reported in various tumors, including lung and prostate cancers. Gomes et al. found that over-expression of MCT4 is associated with poor prognosis in prostate cancer.[19] Gallagher et al. illustrated that, *in vitro*, silencing of MCT4 results in decreased cancer cell migration by mechanisms that involve interaction of MCT4 with integrin β1.[30] However, another study by Izumi et al. showed that silencing of MCT1 or MCT4 inhibited cancer cell invasion, but did not affect cell migration.[9] In the present study, we found high expression of MCT4 in OSCC patients by using IHC. The over-expression of MCT4 was closely associated with tumor size, TNM classification, lymphatic metastasis, distant metastasis, and tumor recurrence. Furthermore, we demonstrated the prognostic significance of MCT4 in OSCC patients. We found that the MCT4 expression level was inversely correlated with both OS and DFS by the Kaplan–Meier method. Patients with higher expression of MCT4 had a reduced survival time. In a multivariate survival analysis, we found that high MCT4 expression was a significant predictor for poor clinical outcome in OSCC patients. These findings suggest that MCT4 might act as a reliable prognosis predictor for individual patients of OSCC.

To further investigate its role in OSCC, we depleted MCT4 expression in OSCC cell lines using siRNA. As is reported that lactate could also been transported via MCT1, which enables lactate entry into cells [31]. We therefore investigated whether the expression of MCT1 could compensate for the knock down of MCT4. We found that the expression of MCT1 was not affected, thus may not compensate for the deficiency of MCT4 in HN4 and HN6 cells. As MCT4 provides a pivotal role in lactate efflux, the concentration of lactate released from single cells was then measured to evaluate MCT4 function. And as expected, when MCT4 was knocked down, lactate release was significantly decreased. Consistent with the close associations between MCT4 expression with tumor size and lymphatic and distant metastasis that was observed in our clinical samples, we found that depletion of MCT4 hindered tumor cell proliferation, migration, and invasion, which indicates that suppression of MCT4 might reverse tumor progression and metastasis, making it a potentially effective therapeutic target.

It has been reported that interaction of MCT4 with integrin is associated with cell migration in vitreoretinopathy, which we confirmed in the present study.[30] Indeed, the expression of integrin β4 was suppressed by MCT4 silencing. We also found a correlation between MCT4-induced integrin β4 inhibition and the down-regulation of SRC-FAK and MEK-ERK signaling pathways. In both HN4 and HN6 cells, depletion of integrin β4, induced by MCT4 silencing, blocked the phosphorylation of FAK-Tyr397, FAK-Tyr925, SRC, MEK, and ERK1/2 without affecting the total levels of FAK, SRC, MEK, or ERK1/2. Integrins are known to function as cell-adhesion receptors that mediate interactions of cells with the extracellular matrix.[32] Several β-integrins including β1, β3, β4, and β5 have been linked to invasion, EMT, and cancer stem cell biology.[33], [34], [35] Their downstream pathways or molecules are different and include FAK, Src kinase, integrin-linked kinase, PI3K/Akt, and mitogen activated protein kinase.[36] Integrins bind FAK and activate FAK phosphorylation at several Tyr residues including Y397, Y861, and Y925, thereby contributing to focal adhesion. The autophosphorylation of FAK on Y397 creates a high-affinity binding site for SRC.[37] When SRC binds to FAK, [38], [39] the integrin-FAK-SRC signaling complex recruits a number of signaling proteins such as those of the MEK-ERK axis,[40], [41], [42] which is involved in adhesion regulation, and the motile and invasive phenotype, as well as in growth and survival signaling.[37], [43] Our data suggest that the restrained intracellular link of integrin β4 to SRC-FAK and MEK-ERK induced by MCT4 silencing might explain the decreased proliferation, migration, and invasion ability observed in OSCC cell lines.

It has been reviewed by Thiery et al, that EMT is a potential mechanism for carcinoma progression.[25] During EMT, cell–cell adhesion molecules, such as E-cadherins and γ-catenin, are down-regulated [44] making it easier for tumor cells to detach from one another and to migrate into the extracellular matrix.[45] Evidence has shown that phosphorylation at FAK-Y925 is the major SRC-specific phosphorylation event associated with integrin adhesion dynamics and E-cadherin deregulation during SRC-induced EMT.[24] In the present study, we show that with MCT4 knock-down, E-cadherin increased and N-cadherin decreased, which prevented tumor cell migration in OSCC cell lines. This further supports a role for MCT4 in the regulation of migration and invasion ability of OSCC cells.

In summary, this study demonstrated that MCT4 is closely correlated with cell proliferation, migration, and invasion ability in OSCC, and it is a poor prognosis predictor for OSCC patients. MCT4 mediates cell proliferation may *via* two major proliferation pathways, AKT and MEK-ERK. The decrease of intracellular signaling of integrin β4 to SRC-FAK and MEK-ERK signaling after knock down of MCT4, may account for the decreased cell migration and invasion. This study also suggested that MCT4 may be a potential metabolic target for the treatment of OSCC in future.
